# A Case Report of Somnambulism Associated With Olanzapine

**Published:** 2012

**Authors:** Farhad Faridhosseini, Azar Zamani

**Affiliations:** 1 Psychiatry and behavioral Sciences Research Center, Ibn-e-Sina Hospital, Faculty of Medicine, Mashhad University of Medical Sciences, Mashhad, Iran; 2 Psychiatry and behavioral Sciences Research Center, Imam Hossein General Hospital, Shahid Beheshty Medical University, Tehran, Iran.

**Keywords:** Olanzapine, Sleepwalking, Somnambulism

## Abstract

Somnambulism consists of a group of behaviors leading to unwanted movements during sleep or even sleepwalking. Medications applied for psychiatric disorders could increase the likelihood of somnambulism in adults. The following article is a case report of somnambulism seen in a patient with schizophrenia, which occurred after remission of an acute episode following treatment with olanzapine. When olanzapine dosage was decreased, no previous and similar symptoms were reported after 6 months of follow up.

## Introduction

Parasomnias are a category of sleep disorders which involves arousal, abnormal and unpleasant experiences in emotions, behaviors and movements. One disorder belonging to this category is somnambulism (1). Somnambulism consists of a group of behaviors leading to unwanted movements during sleep or even sleepwalking, which occurs in children of 4-8 years of age and disappears by teenage years (2,3,4)^.^ Patients with somnambulism wake up doing some motor acts such as walking, running, talking, screaming, while they are confused and they don’t have any relocation of their behavior for the next day(5). Somnambulism may be the result of drug side-effects, psychological stress, unusual breathing during sleep or fever (2).

Medications applied for psychiatric disorders may increase the likelihood of somnambulism in adults (4). Sleepwalking is mostly seen when alcohol is consumed with anti-psychotic and sedative medications, anti-depressants, antihistamines and stimulants (2). Psychiatric disorders such as depression, bipolar disorder and schizophrenia are also associated with parasomnias and they may increase the chance of sleepwalking (4). The following article is a case report of somnambulism seen in a patient with schizophrenia, which occurred after remission of an acute episode following treatment with olanzapine.

## Case Report

The patient was a 42 year old male, who had symptoms of psycho-motor agitation, auditory hallucinations, self-talking, changes in appetite and sleep, therefore was hospitalized in a psychiatric ward. He was diagnosed with schizophrenia and went under treatment with first generation antipsychotic drug, haloperidol. The patient stopped taking haloperidol due to extra pyramidal side effects two weeks after starting treatment; then olanzapine 5mg was prescribed and increased to 20 mg per day. He was discharged 40 days after admission because of symptoms improvement. Patient came for his follow-up monthly. Three months after discharging, reports of sleepwalking with open eyes and loud talking were given by his wife. She reported that the patient had been relocating his cloths and personal belongings in his room, while he had not been able to reply his wife. He had got back to bed and continued sleeping, while having no memory of the event the next day. He had no delusions and hallucinations and he was relatively functional. The patient had no personal or family history of somnambulism. He didn’t have the history of taking alcohol, nicotine or any other illegal drugs. He was prescribed clonazepam 1mg per night and olanzapine dosage was decreased from 20mg daily to 15mg. On his next monthly visit, no previous symptoms were reported by his wife and no similar symptoms were reported after 6 months of follow up.

## Discussion

Sleep disorders such as restless leg syndrome, sleep apnea, sleepwalking and eating while sleeping could all be considered as the side-effects of anti-psychotic medications (6,7). Somnambulism occurs during slow wave sleep period, or the first third of the night. It can be caused by induced arousal during the stage III and IV of the sleep leading to abnormal motor acts without complete awareness (1, 8). Previous studies on pathogenesis of somnambulism indicated enhanced serotonergic activities. Atypical anti-psychotics, including olanzapine and risperidone, increase slow wave sleep via blockade of serotonin receptors (5-HT2C), which leads to sleepwalking (9, 10). As previously mentioned, the patient had no history of somnambulism. Prescribed olanzapine led to walking and talking during sleep with no memory of the event next day.

In a report presented by Kolivakis et al, olanzapine caused somnambulism in two schizophrenic patients, which was no longer observed after patients stopped taking the medication (8).Moreover, Chiu et al reported somnambulism in a bipolar patient following the use of olanzapine, which was still continued after drug dosage, was decreased. In this case, somnambulism only stopped when medication was no longer taken by the patient (3).

Other precipitating factors for somnambulism in adults should be considered. Somnambulism may occur in 1 to 4 % of adults; hence most of cases are continuation of childhood behavior, which can be activated by medication or occurrence of a kind of psychopathology (4). In our case, there is no report of parasomnias in his personal or familial history; however it may be because of recalling bias. Non-antipsychotic medications (e.g. lithium) and substances such as opioids, nicotine and alcohol can increase slow wave sleep, induce nocturnal arousal and lead to somnambulism and other arousal disorders of sleep (7-13). Substance use history in the present case was negative.

The most common treatment for sleep disorders of non-REM stage is benzodiazepine, with clonazepam being the most prescribed medication (4,7,11)^.^ Melatonin (3–15 mg qhs) and pramipexole (0.5–1 mg qhs) may be effective. They can especially used in patients who cannot tolerate benzodiazepines because of its complications or may have a history of substance abuse or dependence (14,15)^.^ The patient in our case report was given clonazepam, olanzapine dosage was decreased and somnambulism was discontinued.

Other atypical anti-psychotics may as well increase slow wave sleep by means of 5-HT2C serotonin receptor blockade, leading to various sleep disorders (9,10). Preueter et al reported two cases of pavor nocturnus (or night terror) due to single nightly dose of risperidone. This happened during the first third of the night and characterized by waking abruptly, accompanied with screaming, gasping and running around room. The symptoms were resolved after dividing medication dosage into two per day.^12^ Increase in slow wave sleep by the use of risperidone was thought to be the cause of night terror; and the symptoms disappeared when drug dosage was divided, which led to decreased level of drug in plasma (12).

Parasomnias are found in 30% to 80% of patients with schizophrenia (6,9). Atypical anti-psychotic medications may enhance patients’ REM latency and slow wave sleep, resulting in improved rest.^6,9 ^However, side-effects are known as somnambulism and other parasomnias, which requires the attention of health care professionals to this issue (6).

## Authors’ Contributions

AZ wrote the first manuscript, based on a clinical case managed by FF. The first draft was revised by FF. Both authors read and approved the final manuscript.

## Notes

**Declaration of interest:** None
